# Supplementary material for: Efficacy and safety of mirror therapy for post-stroke aphasia: A systematic review and meta-analysis protocol
Source: PLoS One. 2024 May 8;19(5):e0301468. doi: 10.1371/journal.pone.0301468 (PMC11078370; doi:10.1371/journal.pone.0301468)
Supplement: S1 Table — (DOC) [file pone.0301468.s001.doc]

| Table 1 search strategy used in PubMed database. | |
| --- | --- |
| no. | search items |
| #1 | Aphasia [MeSH] |
| #2 | Alogias [Title/Abstract] |
| #3 | Logasthenia [Title/Abstract] |
| #4 | Anepia [Title/Abstract] |
| #5 | Global Aphasia [Title/Abstract] |
| #6 | Dysphasia [Title/Abstract] |
| #7 | Word Deafness [Title/Abstract] |
| #8 | #1 or #2-7 |
| #9 | Strokes [MeSH] |
| #10 | Cerebrovascular Accident [Title/Abstract] |
| #11 | Apoplexy [Title/Abstract] |
| #12 | Cerebral Stroke [Title/Abstract] |
| #13 | Brain Vascular Accidents [Title/Abstract] |
| #14 | Cerebrovascular Stroke [Title/Abstract] |
| #15 | #9 or #10-14 |
| #16 | Mirror Movement Therapy [Title/Abstract] |
| #17 | Mirror Movement Therapies [Title/Abstract] |
| #18 | Mirror Therapy [Title/Abstract] |
| #19 | mirror box [Title/Abstract] |
| #20 | visual mirror feedback [Title/Abstract] |
| #21 | #16 or #17-20 |
| #22 | Randomized controlled trial (all field) |
| #23 | Controlled clinical trial (all field) |
| #24 | Randomized (all field) |
| #25 | Random allocation (all field) |
| #26 | Randomly (all field) |
| #27 | Placebo (all field) |
| #28 | Double-blind method (all field) |
| #29 | Single-blind method (all field) |
| #30 | Trials (all field) |
| #31 | #22 or #23-30 |
| #32 | #8 and#15 and #21 and #31 |
