# Supplementary material for: Efficacy and safety of mirror therapy for post-stroke aphasia: A systematic review and meta-analysis protocol
Source: PLoS One. 2024 May 8;19(5):e0301468. doi: 10.1371/journal.pone.0301468 (PMC11078370; doi:10.1371/journal.pone.0301468)
Supplement: Attachment — Submitted filename: a rebuttal letter.docx [file pone.0301468.s003.docx]

**Manuscript:**(PONE-D-23-24347)

**Title:** Efficacy and safety of mirror therapy for post-stroke aphasia: A systematic review and meta-analysis protocol

**A Rebuttal Letter**

Reviewer # 1:

**Comment 1:** Please provide a correct definition of aphasia. Aphasia is a language disorder and speech can be affected in aphasia as aphasia can co-occur with apraxia and dysarthria. "Aphasia is characterized by slowed speech, distorted phonemes, and a tendency for speech to separate into individual syllables with balanced stress between adjacent syllables [4]". This is an incorrect definition of aphasia. These are features of apraxia and dysarthria. Aphasia is a language disorder and there are different types of aphasia. For this protocol, it appears that the authors are discussing about non-fluent aphasia.

**Response:** Thanks for pointing out the mistake. We have corrected the misdescription of aphasia and literature citations as follows “post-stroke aphasia is an acquired language disorder in which patients have impairments in all aspects of the language system [3], mainly including difficulties in auditory comprehension, spontaneous speech, repetition and naming [4]. Different patients have different manifestations of aphasia. Even within the same patient, symptoms related to aphasia can vary considerably, especially in the first weeks and months after stroke. The different types and severity of aphasia depend on many factors, including the size and location of the stroke, health status, time to recovery from stroke, and time since stroke [5].”

**Additional references:**

3.	Sheppard SM, Sebastian R. Diagnosing and managing post-stroke aphasia. Expert review of neurotherapeutics. 2021;21(2):221-34. Epub 2020/11/25. doi: 10.1080/14737175.2020.1855976. PubMed PMID: 33231117; PubMed Central PMCID: PMCPMC7880889.

5.	Basilakos A, Fillmore PT, Rorden C, Guo D, Bonilha L, Fridriksson J. Regional white matter	damage predicts speech fluency in chronic post-stroke aphasia. Frontiers in human neuroscience. 2014;8:845. Epub 2014/11/05. doi: 10.3389/fnhum.2014.00845. PubMed PMID: 25368572; PubMed Central PMCID: PMCPMC4201347.

The deleted quotes are as follows “Aphasia is characterized by slowed speech, distorted phonemes, and a tendency for speech to separate into individual syllables with balanced stress between adjacent syllables [4]. This aphasia is usually caused by damage to the speech area in the anterior left hemisphere of the brain [5]”.

**References that were removed:**

4.	Maas E, Robin DA, Austermann Hula SN, Freedman SE, Wulf G, Ballard KJ, et al. Principles of motor learning in treatment of motor speech disorders. American journal of speech-language pathology. 2008;17(3):277-98. Epub 2008/07/30. doi: 10.1044/1058-0360(2008/025). PubMed PMID: 18663111.

5.	Papale AE, Hooks BM. Circuit changes in motor cortex during motor skill learning. Neuroscience. 2018;368:283-97. Epub 2017/09/18. doi: 10.1016/j.neuroscience.2017.09.010. PubMed PMID: 28918262; PubMed Central PMCID: PMCPMC5762136.

**Comment 2:** Please provide a clear rationale as to why mirror therapy may benefit patients with aphasia. Mirror therapy was originally developed for improving motor function.

**Response**: Thank you for your question, this question is very meaningful, I will answer from two aspects.

1. Mirror therapy, along with action observation therapy and motor imagery therapy, is one of the mirror neuron system therapies. The mirror neuron system (MNS) has been implicated in the development of language and speech, empathy, theory of mind, and social communication [1]. Furthermore, the discovery of a mirror neuron system provides novel neurobiological support for the idea that language and gesture share a common neural substrate [2]. It has been founded that MNS to be involved in the process of gestural communication as well as speech and language functions[3, 4] and to be shared by observation and execution of arm or mouth/lip actions and spoken language [2, 5]. There have been good reports [6, 7] of mirror therapy and action observation therapy that based on MNS for post-stroke aphasia. Therefore, it can be considered that mirror therapy is beneficial to aphasia after stroke.

2. Compared with action observation therapy and motor imagery therapy, mirror therapy puts more emphasis on ontological motor behavior. There is ample agreement in the scientific literature, across diverse areas of study, that suggests that language and movement are interrelated. In particular, it is widely held that the upper limb and hand play a key role in language use [8]. Thus, the combined hand-arm-language paradigm of mirror therapy combined with speech rehabilitation may prove beneficial for aphasia recovery in stroke patients.

**References:**

1.	Chen W, Yuan TF. Mirror neuron system as the joint from action to language. Neuroscience bulletin. 2008;24(4):259-64. Epub 2008/08/01. doi: 10.1007/s12264-008-1231-y. PubMed PMID: 18668155; PubMed Central PMCID: PMCPMC5552584.

2.	Gentilucci M, Dalla Volta R. Spoken language and arm gestures are controlled by the same motor control system. Quarterly journal of experimental psychology (2006). 2008;61(6):944-57. Epub 2008/05/13. doi: 10.1080/17470210701625683. PubMed PMID: 18470824.

3.	Fadiga L, Craighero L, Buccino G, Rizzolatti G. Speech listening specifically modulates the excitability of tongue muscles: a TMS study. The European journal of neuroscience. 2002;15(2):399-402. Epub 2002/02/19. doi: 10.1046/j.0953-816x.2001.01874.x. PubMed PMID: 11849307.

4.	Tettamanti M, Buccino G, Saccuman MC, Gallese V, Danna M, Scifo P, et al. Listening to action-related sentences activates fronto-parietal motor circuits. Journal of cognitive neuroscience. 2005;17(2):273-81. Epub 2005/04/07. doi: 10.1162/0898929053124965. PubMed PMID: 15811239.

5.	Ross LA, Saint-Amour D, Leavitt VM, Javitt DC, Foxe JJ. Do you see what I am saying? Exploring visual enhancement of speech comprehension in noisy environments. Cerebral cortex (New York, NY : 1991). 2007;17(5):1147-53. Epub 2006/06/21. doi: 10.1093/cercor/bhl024. PubMed PMID: 16785256.

6.	Chen W, Ye Q, Ji X, Zhang S, Yang X, Zhou Q, et al. Mirror neuron system based therapy for aphasia rehabilitation. Frontiers in psychology. 2015;6:1665. Epub 2015/11/19. doi: 10.3389/fpsyg.2015.01665. PubMed PMID: 26579046; PubMed Central PMCID: PMCPMC4627352.

7.	Chen Q, Shen W, Sun H, Shen D, Cai X, Ke J, et al. Effects of mirror therapy on motor aphasia after acute cerebral infarction: A randomized controlled trial. NeuroRehabilitation. 2021;49(1):103-17. Epub 2021/06/29. doi: 10.3233/nre-210125. PubMed PMID: 34180428.

8.	Wortman-Jutt S, Edwards D. Poststroke Aphasia Rehabilitation: Why All Talk and No Action? Neurorehabilitation and neural repair. 2019;33(4):235-44. Epub 2019/03/23. doi: 10.1177/1545968319834901. PubMed PMID: 30900528.

**Comment 3:** The primary and secondary outcomes as assessing aphasia as a global measure ( e.g., WAB, BDAE etc). It is also important to determine whether mirror therapy has an effect on quality of life or functional communication.

**Response:** Thank you very much indeed for your comments. I added Communication Activities of Daily Living (CADL), Functional Assessment of Communication Skills (FACS) and Hamilton Depression Scale (HAM-D) in “Types of outcomes”.

Reviewer # 2:

**Comment 1:** In the section “2.1.1 Types of participants” the age group of the patients to be included is not mentioned. Throughout the text, age of patients is only mentioned in the following paragraph of the discussion “The effectiveness and safety of MT in adult patients with post-stroke aphasia will thus be the focus of the first systematic review and meta-analysis in this field”. I suggest clarifying this point.
**Response:** Thank you very much indeed for your comments. As you mentioned, I added the age group in the "2.1.1 Types of Participants" section to be patients 18 years of age or older.

**Comment 2:** In the first paragraph of the discussion, you mention the number of patients with aphasia secondary to stroke and traumatic brain injury, however, patients with trauma will not be included in this study, so I suggest that the text refers only to stroke patients.

**Response:** Thank you very much indeed for your comments. In the first paragraph of the discussion, I have removed the description of traumatic brain injury and added a description of the epidemiology of post-stroke aphasia. The details are as follows “aphasia is common following stroke, with frequencies ranging from 15 to 42 percent in acute settings and from 25 to 50 percent in chronic settings. [9]”.

**Additional references:**

9.	Flowers HL, Skoretz SA, Silver FL, Rochon E, Fang J, Flamand-Roze C, et al. Poststroke Aphasia Frequency, Recovery, and Outcomes: A Systematic Review and Meta-Analysis. Archives of physical medicine and rehabilitation. 2016;97(12):2188-201.e8. Epub 2016/04/12. doi: 10.1016/j.apmr.2016.03.006. PubMed PMID: 27063364.
